# Design and implementation of a mobile system for lung cancer patient follow-up in China and initial report of the ongoing patient registry

**DOI:** 10.18632/oncotarget.13720

**Published:** 2016-11-30

**Authors:** Xiangyun Ye, Jia Wei, Ziming Li, Xiaomin Niu, Jiemin Wang, Yunqin Chen, Zongming Guo, Shun Lu

**Affiliations:** ^1^ Shanghai Lung Cancer Center, Shanghai Chest Hospital, Shanghai Jiaotong University, Shanghai, China; ^2^ Research and Development Information China, AstraZeneca, Pudong, Shanghai, China

**Keywords:** lung cancer, China, mobile app, registry, data integration

## Abstract

**Introduction:**

Management of lung cancer remains a challenge. Although clinical and biological patient data are crucial for cancer research, these data may be missing from registries and clinical trials. Biobanks provide a source of high-quality biological material for clinical research; however, linking these samples to the corresponding patient and clinical data is technically challenging. We describe the mobile Lung Cancer Care system (mLCCare), a novel tool which integrates biological and clinical patient data into a single resource.

**Methods:**

mLCCare was developed as a mobile device application (app) and an internet website. Data storage is hosted on cloud servers, with the mobile app and website acting as a front-end to the system. mLCCare also facilitates communication with patients to remind them to take their medication and attend follow-up appointments.

**Results:**

Between January 2014 and October 2015, 5,080 patients with lung cancer have been registered with mLCCare. Data validation ensures all the patient information is of consistently high-quality. Patient cohorts can be constructed *via* user-specified criteria and data exported for statistical analysis by authorized investigators and collaborators. mLCCare forms the basis of establishing an ongoing lung cancer registry and could form the basis of a high-quality multisite patient registry. Integration of mLCCare with SMS messaging and WeChat functionality facilitates communication between physicians and patients.

**Conclusion:**

It is hoped that mLCCare will prove to be a powerful and widely used tool that will enhance both research and clinical practice.

## INTRODUCTION

Lung cancer remains a global public health problem, with high prevalence, poor prognosis, and high treatment costs [1–4]. In 2012, there were approximately 1.82 million new diagnoses of lung cancer worldwide and nearly 1.6 million deaths [5, 6]. Thus, management of lung cancer remains a major public health challenge [7–9].

Patient registries can provide important information with respect to disease epidemiology, prevalence rates, treatment practices, and patient outcomes. Information, such as response to targeted therapies and biomarker data, is highly valuable for cancer research. However, these data may be limited or missing.

In China, national cancer registries have been established and are currently still maturing [10–12]. Existing Chinese registries tend to be regional and the majority of hospitals only retain patient records for the duration of the patient's life or period of hospitalization. As a result, patients’ data (i.e. treatment, side-effects, epidemiology, and outcome) may be lost or destroyed with time. Overall, in many Chinese hospitals there remains no standardized method of storing and utilizing clinical data.

In addition to clinical and epidemiologic data, information from a patient's biological sample (as well as the actual samples themselves) represent a rich source of data; however, long-term storage may be an issue. Biobanking allows the storage of high-quality tissue samples which can be used for diagnostic, prognostic, and research purposes, including biomarker discovery and validation of putative therapeutic targets [13]. However, linking biobank samples to the corresponding patient information and clinical data is complex and requires the integration of numerous data management systems [14]. The integration of biobank databases with patient clinical data therefore remains a significant unmet need.

Recent advances in computer and mobile communications technology have been utilized by clinics and hospitals to monitor and manage patients with cancer [15]. Utilizing these technologies may allow information from multiple biobanks and patient databases, including patient clinical information and biological samples, to be integrated into one system to allow long-term follow-up and analysis.

We have developed a novel system, mobile Lung Cancer Care (mLCCare), to facilitate the integration of epidemiologic, treatment, and outcomes data for patients with lung cancer in China. Stored data also includes information on tumor biomarkers and biomarker changes over time. mLCCare also connects to a biobank database, thus linking biological and clinical data together into one single, powerful resource. Here we describe the development of mLCCare and the initial report on the registry.

## MATERIALS AND METHODS

### mLCCare architecture

mLCCare was developed as a mobile application (app) and internet website. All operations and data storage are hosted on cloud servers with the mobile app and website acting as a front-end to the system. mLCCare was developed using Java 2 Platform, Enterprise Edition (Oracle Corp., California, USA), to create a web-based three-tier application. The first tier is the end-user web interface and mobile application. The second tier contains the application's business logic to manage the workflow and data handling. The third tier is an integration tier that consists of the enterprise resources. mLCCare runs on a NGINX platform (NGINX, Inc., California, USA) as a reverse proxy to manage resources between the client software and the web servers, with a MySQL (Oracle Corp., California, USA) cluster as the back-end database.

### Data pathway

mLCCare was designed to allow the integration of patients’ clinical, biobank, and biomarker data. Furthermore, the system facilitates communication with patients to provide reminders for taking medication and follow-up appointments (Table 1). The system was developed to be used with both computer-based internet browsers (Internet Explorer 8 and above, or other major browsers) and mobile apps (both Apple iOS [version 6 or above] and Google Android OS [version 4 or above]).

**Table 1:** Key users of mLCCare

User	Role/Responsibility
Physicians	Management of lung cancer patients and input of clinical data
Principle investigators	Oversight of all patients registered on mLCCare
Research collaborator	Analysis of collected data, with permission from principle investigators
Administrator	System and database management
Patients	End-users who receive communications via SMS message and WeChat

Abbreviation: mLCCare, mobile Lung Cancer Care.

**Figure 1:**
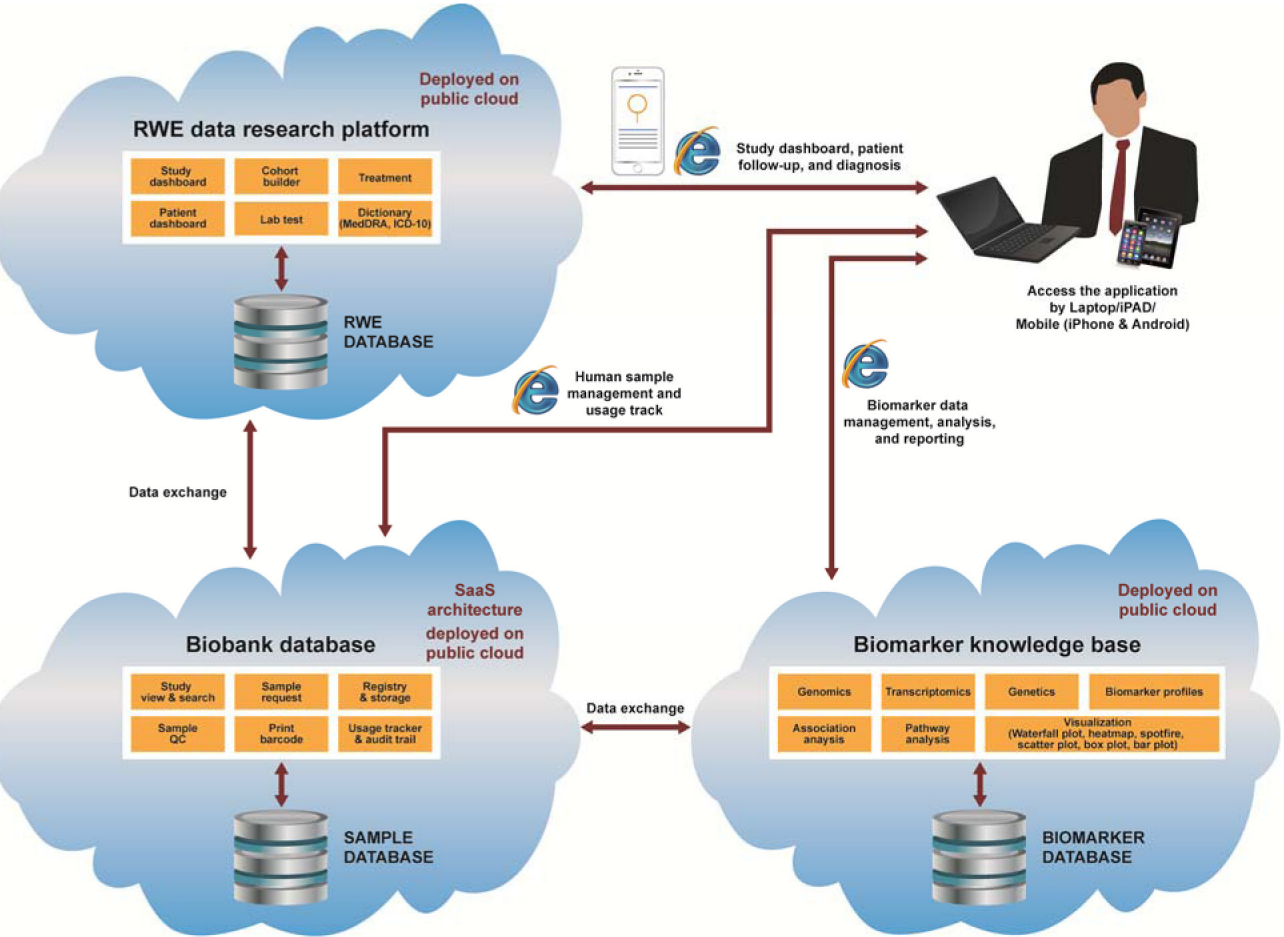
ICD-10, 10 ^th^ revision of the International Statistical Classification of Diseases and Related Health Problems; MedDRA, Medical Dictionary for Regulatory Activities; PAD, personal access device; QC, quality control; RWE, real-world evidence; SaaS, software as a service.

mLCCare was developed to collect, store, and share information (Table 2). Clinical data collected by physicians during consultations with patients can be added to the patient record *via* a computer using the website or *via* the app using iOS or Android phones and tablets. These data are stored on the cloud-based platform (Figure 1). Clinical and patient data inputs were designed to be simple, with most fields being completed with dropdown boxes or checkboxes.

**Table 2:** Clinical and biomarker data collected from patients via mLCCare

Category	Parameters	Example
General	Age	
	Sex	
	Hospitalization times	
Diagnosis	Histological type	NSCLC, small cell carcinoma
	Differentiation	Well, poor
	Stage (TNM)	I, IIA, IIB, III, IV
	Lymph nodes detected in surgery	
	Clinical symptoms	Cough, chest pain, dyspnea, etc.
	Others	CT scan, MRI scan, PET scan, etc.
	Biopsy	Lymph nodes, hydrothorax, etc.
Biomarker	Biomarker	EGFR mutations, ALK fusion, ROS-1, etc.
Treatment	Surgery type	Wedge resection, lobectomy, etc.
	Chemotherapy (regimen, drug, dosage)	Paclitaxel, cisplatin, docetaxel, pemetrexed, etc.
	Target therapy	Gefitinib, Erlotinib, Icotinib, Crizotinib, etc.
	Radiotherapy	
Disease progression	Metastasis	Liver, brain, lymph nodes
	Recurrence	
Treatment outcomes	Response	Partial response, complete response, progress disease, etc.
	Symptomatic response	Complete disappearance, good improvement, no change, worsening, etc.
Treatment side-effects	Organ-based toxicity	Skin, hair loss, liver, kidney, etc.
	Bone marrow suppression	
Quality of life	Quality of life	KPS rating (0–100)

Abbreviations: ALK, anaplastic lymphoma kinase; CT, computed tomography; EGFR, epidermal growth factor receptor; KPS, Karnofsky Performance Status; mLCCare, mobile Lung Cancer Care; NSCLC, non-small cell lung cancer; PET, positron emission tomography; TNM, primary tumor characteristics, nearby lymph node involvement, status of distant metastases.

In addition to the clinical data collected by physicians during consultations with patients, mLCCare links patient records with their respective biomarker information (such as epidermal growth factor receptor [EGFR] mutation status) in the biomarker knowledge base and also links to biobank tissue samples. These data are also stored on cloud servers. The biomarker knowledge base collates and provides information regarding the genomics, transcriptome, and biomarker profile of patients. The biobank database permits viewing of samples, sample requests, registry and storage status, sample quality control status, and tracking and auditing of sample use (Supplementary Figure S1).

### Data validation, usage, and analysis

Data quality measures have been built in to the architecture of mLCCare to ensure the fidelity of the data recorded in the registry. User input is validated on both the server-end and client-end. Validation components have been built in to the application, including a JavaScript validator to ensure fields are filled in correctly.

A query management system, source data validation, provides an additional level of quality assurance. If an error is detected in an input field a query is generated prompting users to correct the error or indicating that there is no error if, for example, a nonstandard answer has been given in one field.

mLCCare allows authorized users to analyze data in real-time and generate reports of patients’ clinical progress over time, both for individual patients and for user-specified cohorts. Parameters include response to therapy, disease progression, recurrence, the acquisition of resistance (linked to biomarker data) and overall survival. This permits analysis by physicians and authorized researchers. In addition, the biomarker knowledge base provides tools for association analyses, pathway analyses, and data visualization.

### Data security

mLCCare is only available to registered and authorized users. Each user is assigned a unique identifier and password. Password aging has been implemented to prompt regular password changes, increasing overall security and limiting the potential for unauthorized access to the system. To maximize security, passwords are required to be a minimum of eight characters, with a mix of lower- and upper-case letters, special characters, and numbers.

Each user has a specific level of privilege and access to the data. Researchers are permitted to view de-identified and anonymized data that they have been granted permission to. Physicians are able to add, view, edit, or delete data of their own patients. Study owners can view all of the data of patients who are enrolled in studies overseen by the user.

All patient data held in mLCCare is de-identified or anonymized when entered into the database. De-identification removes or replaces personal identifiers associated with patient data, making it difficult to link an individual and their data. Anonymization irreversibly removes the link between the individual patient and their data, making it virtually impossible to re-establish the link. This ensures that patient confidentially and anonymity is preserved.

To achieve the highest levels of security, only specific internet protocol (IP) addresses are permitted to connect to the Linux virtual private server *via* Secure Socket Shell. This ensures that, in addition to authorized users, only authorized devices can connect to mLCCare. Furthermore, the database security settings provide additional security. The database is executed in a secured environment and all database processes run under a unique identifier that is not shared by any other system processes. All example database instances and tables in the system are removed. Database access is limited by device and anonymous access to the database is not permitted. The administrator account does not use a default name and the database root's account is password protected.

mLCCare utilizes Hypertext Transfer Protocol Secure (HTTPS) for web server communication. HTTPS is a combination of the Hypertext Transfer Protocol (HTTP) with the Secure Socket Layer (SSL) protocol to provide encrypted communication and secure identification of a network web server. We established MySQL master-slave replication for load-balance and backup. Replication enables data from one MySQL database server (the master) to be replicated to one or more additional servers (the slaves). Because data is replicated to the slave(s), it is possible to run backup services on the slave(s) without corrupting the corresponding master data.

### Data integrity

Audit trail functionality is built in to mLCCare to ensure data integrity. The audit trail feature records the identity of users entering, changing, confirming, or deleting any data in the system, and time-stamps this information.

### mLCCare interface

As previously described, the mobile apps for mLCCare have been designed to operate on iOS (version 6 and above) and Android (version 4 and above) mobile devices. An internet browser interface is also available at http://rwe.rwebox.com/rwe-web/login.html which has been designed to closely replicate the interface in the iOS and Android applications (Supplementary Figure S2).

### Patient communication facilities

The mobile application for mLCCare has been developed with integrated SMS messaging and WeChat functionality to provide appointment reminders. Reminders can be sent from physicians to patients one day prior to their scheduled appointment.

### Enrollment

mLCCare has been available to participating physicians since January 2014.

### Biomarker testing

Amplification-refractory mutation system (ARMS) PCR was used to detect *EGFR*, *KRAS*, *ROS1*, and *BRAF* mutations (16); specifically the ADx-ARMS kit (http://www.amoydiagnostics.com/Gene-Mutation_c1). For anaplastic lymphoma kinase (*ALK*) mutations, the VENTANA ALK (D5F3) CDx assay was used to detect ALK fusions in formalin-fixed, paraffin-embedded non-small cell lung carcinoma (NSCLC) tissue stained with a BenchMark XT automated staining instrument.

### Patient follow-up

After discharge from hospital, patient follow-up can be conducted through WeChat with different requirements during treatment, after treatment completion, or after incidences of adverse events. Questions on quality of life are surveyed at all follow-ups. When a patient is receiving outpatient treatment, they can upload their routine blood test results. The next hospital visit can be scheduled after the physician assesses the test result. After a patient completes treatment, routine exam results (including computed tomography and MRI scan, bone scans, and blood test results) can be uploaded remotely so doctors can monitor disease progression.

## RESULTS AND DISCUSSION

We have described the development of mLCCare, a cloud-based mobile app and website that allows integration of patient records, biomarker data, and biobank samples, as well as providing patients to communicate with their physician. mLCCare allows physicians and investigators to sign in from a computer, smartphone, or tablet and access patient data in one uniform platform. Data can be added when users are offline and synchronized once online. Data validation ensures the quality and consistency of the collected data is maintained by all physicians and investigators. Patient cohorts can be constructed *via* user-specified criteria and data exported for statistical analysis.

mLCCare ensures that all descriptive terms entered are compliant with medical standards. All diagnostic terms are compliant with the 10^th^ revision of the International Statistical Classification of Diseases and Related Health Problems and Medical Dictionary for Regulatory Activities terms, therapeutic terms with Anatomical Therapeutic Chemical classifications and laboratory tests are compliant with Clinical Information Standards Governance Organization standards. Multiple languages, including English, Chinese, and Spanish are currently supported by mLCCare and support for other languages is planned.

The biobank functionality allows users to view, search, and request samples, all of which are linked to the patient-of-origin's clinical data.

### mLCCare as a registry

mLCCare can be used to establish an ongoing and well-maintained lung cancer registry. As all data are updated in real-time by the treating physicians, this represents a potentially powerful resource for registry-based real-world data.

As well as some local or regional cancer registries, national cancer registries, such as the National Central Cancer Registry (NCCR) and the Chinese Multi-Institutional Registry (CMIR) have recently been established [10, 11]. However, the national registry system in China is still maturing and not yet fully established [12]. In comparison with both the NCCR and the CMIR, the registry established through mLCCare has several potential advantages (Table 3).

**Table 3:** Comparison of features offered by mLCCare, NCCR, and CMIR

Features	mLCCare	NCCR	CMIR
Mobile access	+		
Biomarker data	+	+	
Biobank integration	+		
Treatment-emergent adverse events	+		
Disease progression or recurrence	+		
Real-time data updates	+		
SMS or internet messaging	+		

Abbreviations: CMIR, Chinese Multi-Institutional Registry; mLCCare; mobile Lung Cancer Care; NCCR, National Central Cancer Registry.

Currently, mLCCare has been implemented in the Shanghai Lung Cancer Center at Shanghai Chest Hospital. Of the 5,080 patients currently enrolled between January 2014 and October 2015, the majority were aged 50 to 59 years (31.9%), male (58.3%), had stage IV disease (50.6% of 3,546 patients with available staging information), and were diagnosed with non-small cell lung cancer (93.3%) with a pathology of adenocarcinoma (69.5%), (Table 4A). All patients underwent a biopsy at diagnosis and 2,049/5,080 patients were tested for biomarkers (40.3% of all patients). The majority of patients with identifiable mutations were diagnosed with *EGFR*-positive non-small cell lung cancer (44.1%). A summary of the results is shown in Table 4B.

**Table 4A:** Demographics and disease characteristics of enrolled lung cancer patients

Characteristic	n (%) *N* = 5,080
*Age of patients when first enrolled*	
≤40	173 (3.4)
40–49	606 (11.9)
50–59	1,621 (31.9)
60–69	1,927 (37.9)
70–79	722 (14.2)
≥80	31 (0.6)
Average age, years (mean ± SD)	59 (10.0)
*Sex*	
Men^a^	2,960 (58.3)
Women	2,119 (41.7)
*Pathology*	
Small cell carcinoma	341 (6.7)
Non-small cell carcinoma	4,739 (93.3)
Adenocarcinoma	3,531 (69.5)
Squamous cell carcinoma	796 (15.7)
Adenosquamous carcinoma	42 (0.8)
Large cell carcinoma	41 (0.8)
Others^b^	329 (6.5)
*TNM stage (n = 3,546)*^c^	
IA	551 (15.5)
IB	197 (5.6)
IIA	177 (5.0)
IIB	94 (2.7)
IIIA	470 (13.3)
IIIB	261 (7.4)
IV	1796 (50.6)
*Prior treatment*d	
Radiotherapy	667 (13.1)
Surgery	3,000 (59.0)

^a^Data for one patient was not available. ^b^Includes carcinoid tumor, sarcomatoid carcinomas, poorly differentiated tumors and other NSCLC types. ^c^TNM staging was not available for 1,534 patients; ^d^Patients may have received more than one type of treatment.Abbreviations: NSCLC, non-small cell lung cancer; SD, standard deviation.

**Table 4B:** Biomarker profile of enrolled lung cancer patients

*n* (%)	EGFR-positivea	ALK-positive	KRAS-positive	BRAF-positive	ROS1-positive	Positive for at least one biomarkerb
**Small cell carcinoma (*N* = 67)**	26 (1.4)	4 (0.3)	0 (0)	0 (0)	0 (0)	30 (1.5)
**Non-small cell carcinoma** (***N* = 1,982)**	804 (44.1)	121 (8.2)	10 (9.1)	3 (1.4)	27 (4.3)	940 (45.9)
*Adenocarcinoma* *(N = 1,507)*	729 (40.0)	103 (7.0)	9 (8.2)	3 (1.4)	23 (3.7)	846 (41.3)
*Squamous cell carcinoma (N = 325)*	36 (2.0)	8 (0.5)	0 (0)	0 (0)	0 (0)	44 (2.2)
*Adenosquamous carcinoma (N = 19)*	8 (0.4)	1 (0.1)	0 (0)	0 (0)	0 (0)	9 (0.4)
*Large cell carcinoma* *(N = 9)*	2 (0.1)	0 (0)	0 (0)	0 (0)	0 (0)	2 (0.1)
*Others* *(N = 121)*	29 (1.6)	9 (0.6)	1 (0.9)	0 (0)	4 (0.6)	39 (1.9)

^a^%=n/total number of specific biomarker test.

^b^Patients could be positive for more than one biomarker, total number of patients who were analyzed *n* = 2,049.

Of the 5,484 patients, 3,000 patients had surgery for lung cancer and 667 patients received radiotherapy (small cell carcinoma *n* = 98; NSCLC *n* = 569). The most commonly used treatment received, regardless of the type of lung cancer, was platinum-based chemotherapy (Tables 5A and 5B), which remains the standard of care for patients with recurrent disease who are not suitable for treatment with targeted agents, such as EGFR-tyrosine kinase inhibitors (TKIs).[17] EGFR-TKIs were mainly used to treat patients with adenocarcinoma (Table 5A) and were given second- or third-line (Table 5B).

Given the features of mLCCare, we believe it has the potential to form the basis of a detailed patient registry that could be deployed at multiple sites. In principle, the web-based user interface means multiple users can access the service from sites across regions, allowing large-scale and standardized collaboration to build a rich registry. Furthermore, the real-time data updates would provide valuable real-world evidence, a factor that is crucial for patients who tend to have poor prognoses and short survival times.

**Table 5A:** Treatment of enrolled lung cancer patients according to lung cancer type

Overall (*N* = 2,077)	Treatment	Number of patients, *n* (%)
Small cell carcinoma (*N* = 206)^a^	Etoposide, Carboplatin	143 (69.4)
	Etoposide, Cisplatin	60 (29.1)
	Paclitaxel, Carboplatin	21 (10.2)
	Paclitaxel, Nedaplatin	19 (9.2)
	Etoposide	17 (8.3)
	Weekly Irinotecan	17 (4.4)
	Nedaplatin, Etoposide	10 (4.9)
	Paclitaxel	9 (4.4)
	Etoposide, Carboplatin, Endostatin	6 (2.9)
Squamous cell carcinoma (*N* = 406)^a^	Nedaplatin, Docetaxel	146 (35.9)
	Gemcitabine, Carboplatin	94 (23.2)
	Gemcitabine	74 (18.2)
	Docetaxel	64 (15.8)
	Gemcitabine, Cisplatin	59 (14.5)
	Docetaxel, Cisplatin	58 (14.3)
	Vinorelbine, Gemcitabine	43 (10.6)
	Vinorelbine, Carboplatin	34 (8.4)
	Paclitaxel, Carboplatin	29 (7.1)
	Vinorelbine	25 (6.2)
	Nedaplatin, Gemcitabine	25 (6.2)
	S1 (tegafur, gimeracil, and oteracil)	23 (5.7)
	Erlotinib	15 (3.7)
	Docetaxel, Carboplatin	10 (2.5)
	Paclitaxel, Cisplatin	9 (2.2)
Adenocarcinoma (*N* = 1,273)^a^	Pemetrexed, Carboplatin	601 (47.2)
	Gemcitabine, Carboplatin	296 (23.3)
	Pemetrexed	225 (17.7)
	Docetaxel	163 (12.8)
	Gemcitabine	160 (12.5)
	Gefitinib	149 (11.7)
	Icotinib	104 (8.2)
	Pemetrexed, Cisplatin	100 (7.9)
	Vinorelbine, Carboplatin	91 (7.1)
	Gemcitabine, Cisplatin	90 (3.7)
	Erlotinib	79 (6.2)
	Nedaplatin, Docetaxel	60 (4.7)
	Pemetrexed, Nedaplatin	55 (4.3)
	Paclitaxel, Carboplatin	48 (3.8)
	Vinorelbine	46 (3.6)
	Crizotinib	34 (2.7)
	Pemetrexed, Oxaliplatin	33 (2.6)
	Vinorelbine, Cisplatin	33 (2.6)
	S1 (tegafur, gimeracil, and oteracil)	30 (2.4)
	Vinorelbine, Gemcitabine	28 (2.2)

^a^Patients may have received more than one treatment.

**Table 5B:** Summary of treatment by first-, second-, and third-line according to lung cancer type

	First-line (*N* = 64)		Second-line (*N* = 66)		Third-line (*N* = 24)	
Small cell carcinoma	**Treatment**	***n* (%)**	**Treatment**	***n* (%)**	**Treatment**	***n* (%)**
	Etoposide, Carboplatin	47 (60.3)	Etoposide, Carboplatin	43 (42.2)	Etoposide, Carboplatin	6 (17.6)
	Etoposide, Cisplatin	18 (23.1)	Paclitaxel, Nedaplatin	13 (12.7)	Irinotecan	6 (17.6)
	Etoposide	5 (6.4)	Paclitaxel, Carboplatin	12 (11.8)	Paclitaxel, Carboplatin	5 (14.7)
	Lobaplatin, Semustine, Etoposide	2 (2.6)	Etoposide, Cisplatin	11 (10.8)	Docetaxel	3 (8.8)
	Paclitaxel	2 (2.6)	Paclitaxel	8 (7.8)	Nedaplatin, Ifosfamide	3 (8.8)
	Paclitaxel, Carboplatin	2 (2.6)	Weekly Irinotecan	7 (6.8)	Paclitaxel, Nedaplatin	3 (8.8)
	Paclitaxel, Nedaplatin	2 (2.6)	Etoposide	4 (3.9)	Etoposide	2 (5.9)
	-	-	Carboplatin, Abraxane	2 (2.0)	Etoposide, Cisplatin	2 (5.9)
	-	-	Nedaplatin, Etoposide	2 (2.0)	Irinotecan, Cisplatin	2 (5.9)
	-	-	-	-	Paclitaxel, Nedaplatin, Endostatin	2 (5.9)
	**First-line (*N* = 120)**		**Second-line (*N* = 126)**		**Third-line (*N* = 48)**	
	**Treatment**	***n* (%)**	**Treatment**	***n* (%)**	**Treatment**	***n* (%)**
Squamous cell carcinoma	Nedaplatin, Docetaxel	35 (27.8)	Nedaplatin, Docetaxel	42 (18.6)	Docetaxel	15 (18.1)
	Gemcitabine, Carboplatin	24 (19.0)	Docetaxel	33 (14.6)	Nedaplatin, Docetaxel	15 (18.1)
	Docetaxel, Cisplatin	21 (16.7)	Gemcitabine, Carboplatin	24 (10.6)	Vinorelbine, Gemcitabine	14 (16.9)
	Gemcitabine, Cisplatin	21 (16.7)	Docetaxel, Cisplatin	23 (10.2)	S1 (tegafur, gimeracil, and oteracil)	8 (9.6)
	Docetaxel	13 (10.3)	Vinorelbine, Gemcitabine	31 (13.7)	Vinorelbine, Nedaplatin	5 (6.0)
	Paclitaxel, Carboplatin	7 (5.6)	Gemcitabine	19 (8.4)	Erlotinib	4 (4.8)
	Vinorelbine, Carboplatin	5 (4.0)	Gemcitabine, Cisplatin	13 (5.8)	Gemcitabine	3 (3.6)
	–	–	Nedaplatin, Gemcitabine	13 (5.8)	Gemcitabine, Carboplatin	3 (3.6)
	–	–	S1 (tegafur, gimeracil, and oteracil)	7 (3.1)	Nedaplatin, S1 (tegafur, gimeracil, and oteracil)	3 (3.6)
	–	–	Paclitaxel, Carboplatin	5 (2.2)	Pemetrexed	3 (3.6)
	–	–	Erlotinib	5 (2.2)	Abraxane	2 (2.4)
			Vinorelbine, Carboplatin	5 (2.2)	Docetaxel, Cisplatin	2 (2.4)
	–	–	Abraxane	3 (1.3)	Nedaplatin, Gemcitabine	2 (2.4)
	–	–	Paclitaxel	3 (1.3)	Pemetrexed, Nedaplatin	2 (2.4)
	–	–	–	–	Vinorelbine	2 (2.4)
	**First-line (*N* = 333)**		**Second-line (*N* = 399)**		**Third-line (*N* = 176)**	
	**Treatment**	***n* (%)**	**Treatment**	***n* (%)**	**Treatment**	***n* (%)**
Adenocarcinoma	Pemetrexed, Carboplatin	94 (28.2)	Pemetrexed, Carboplatin	131 (18.5)	Docetaxel	61 (20.4)
	Gemcitabine, Carboplatin	89 (26.7)	Pemetrexed	110 (15.5)	Pemetrexed	47 (15.7)
	Gemcitabine, Cisplatin	35 (10.5)	Docetaxel	71 (10.0)	Pemetrexed, Carboplatin	33 (11.0)
	Pemetrexed	32 (9.6)	Gemcitabine, Carboplatin	70 (9.9)	Vinorelbine, Gemcitabine	20 (6.6)
	Pemetrexed, Cisplatin	29 (8.7)	Gefitinib	64 (9.0)	Icotinib	19 (6.4)
	Vinorelbine, Carboplatin	27 (8.1)	Nedaplatin, Docetaxel	32 (4.5)	Gefitinib	18 (6.0)
	Gemcitabine	21 (6.3)	Icotinib	31 (4.4)	Erlotinib	17 (5.7)
	Paclitaxel, Carboplatin	21 (6.3)	Erlotinib	30 (4.2)	Gemcitabine, Carboplatin	13 (4.3)
	Gefitinib	16 (4.8)	Gemcitabine	28 (4.0)	S1 (tegafur, gimeracil, and oteracil)	13 (4.3)
	Vinorelbine, Cisplatin	16 (4.8)	Pemetrexed, Cisplatin	28 (4.0)	Nedaplatin, Docetaxel	11 (3.7)
	Docetaxel	10 (3.0)	Gemcitabine, Cisplatin	22 (3.1)	Pemetrexed, Nedaplatin	10 (3.3)
	Pemetrexed, Nedaplatin	9 (2.7)	Pemetrexed, Nedaplatin	16 (2.3)	Gemcitabine	8 (2.7)
	Docetaxel, Carboplatin	8 (2.4)	Paclitaxel, Carboplatin	15 (2.1)	Pemetrexed, Oxaliplatin	8 (2.7)
	Icotinib	7 (2.1)	Vinorelbine, Carboplatin	15 (2.1)	Crizotinib	5 (1.7)
	–	–	Pemetrexed, Oxaliplatin	11 (1.6)	Docetaxel, Cisplatin	5 (1.7)
	–	–	Docetaxel, Cisplatin	10 (1.4)	Pemetrexed, Cisplatin	5 (1.7)
	–	–	Docetaxel, Carboplatin	8 (1.1)	Afatinib	3 (1.0)
	–	–	S1 (tegafur, gimeracil, and oteracil)	8 (1.1)	Bevacizumab	3 (1.0)
	–	–	Vinorelbine, Gemcitabine	8 (1.1)	–	–

### User experience

Between January 2014 and October 2015, the details of 5,080 patients with lung cancer have been registered on mLCCare. The patient demographics and disease characteristics are given in Table 4A.

Previous examples of mobile apps used for cancer patient management have found that ease-of-use is of central importance for end-users, in particular an intuitive layout of the key user interface elements [18, 19]. Therefore, particular care was given to the design elements of mLCCare to ensure an intuitive and easy-to-learn user interface. The layout was also standardized across all versions (browser, iOS, and Android) to facilitate user comfort with the system. Feedback from physicians who have used the system confirmed that ease-of-familiarization with an application's user interface is of key importance for continued use. To date, feedback has been positive with users finding both the mobile app and website easy to navigate and use. As user-feedback is invited and received, elements of mLCCare may be optimized in accordance with user preferences.

Integration of mLCCare with SMS and WeChat functionality allows communication between physicians and patients and could help to increase treatment compliance and reduce missed clinic visits.

### Data security

Data security was a principal concern in the development of mLCCare. This has been addressed at every level of software development, from user identification, password requirements, user-specific permissions for access to data, database security, and IP address restrictions that maintain control over access to mLCCare.

### Limitations

To date, the mLCCare has only been trialed at the Shanghai Lung Cancer Center, Shanghai Chest Hospital. We expect to increase the number of centers using mLCCare in the near future. Until there is a wider user-base and patients are enrolled from multiple sites, the true potential of mLCCare for establishing a national or international registry is unknown.

The number of patients who received targeted therapies was relatively low. One explanation for this is that most patients were self-funded and so could not afford the cost associated of a biopsy and/or targeted agents, such as EGFR-TKIs.

The integration of social functionality (SMS and WeChat) remains limited. Additional features, such as forums or real-time chat, to facilitate communication between physicians, researchers, and patients may be explored in the future. Remote follow-up of patients *via* WeChat is another feature that could be explored. This may be particularly useful for patients who are unable to attend frequent visits to major hospitals following treatment.

## CONCLUSIONS

Modern communication technology can be utilized to improve both the management of patients and the collection of important clinical data. mLCCare provides a secure, reliable, easy-to-use tool for healthcare professionals to monitor and record patients’ clinical progress, treatment history, and biomarker data. Additionally, these data can be linked to biobank databases, providing a resource of biological samples with linked patient records. In principle this allows for the development of a large patient registry. Such a registry could be important as a research resource. We believe that mLCCare will prove to be a powerful and widely used tool, which will enhance both research and clinical practice.

## SUPPLEMENTARY MATERIALS


