# Supplementary material for: Design and implementation of a mobile system for lung cancer patient follow-up in China and initial report of the ongoing patient registry
Source: Oncotarget. 2016 Nov 30;8(3):5487–97. doi: 10.18632/oncotarget.13720 (PMC5354925; doi:10.18632/oncotarget.13720)
Supplement: Supplementary file 1 [file oncotarget-08-5487-s001.pdf]

Design and implementation of a mobile system for lung cancer patient follow-up in China and initial report of the ongoing patient registry

Supplementary Material

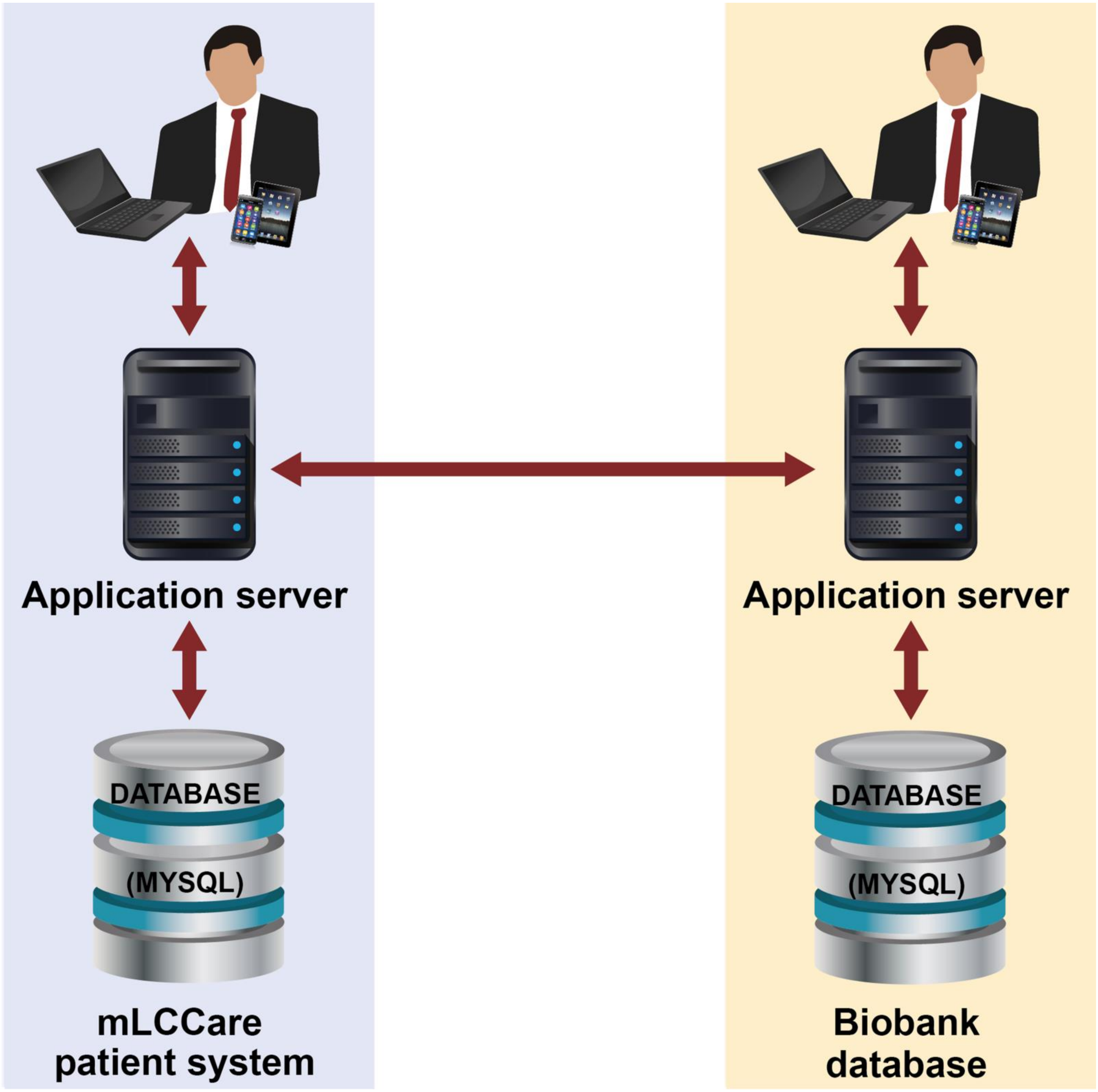

Supplementary Figure S1: Integration of mLCCare and the Biobank Database

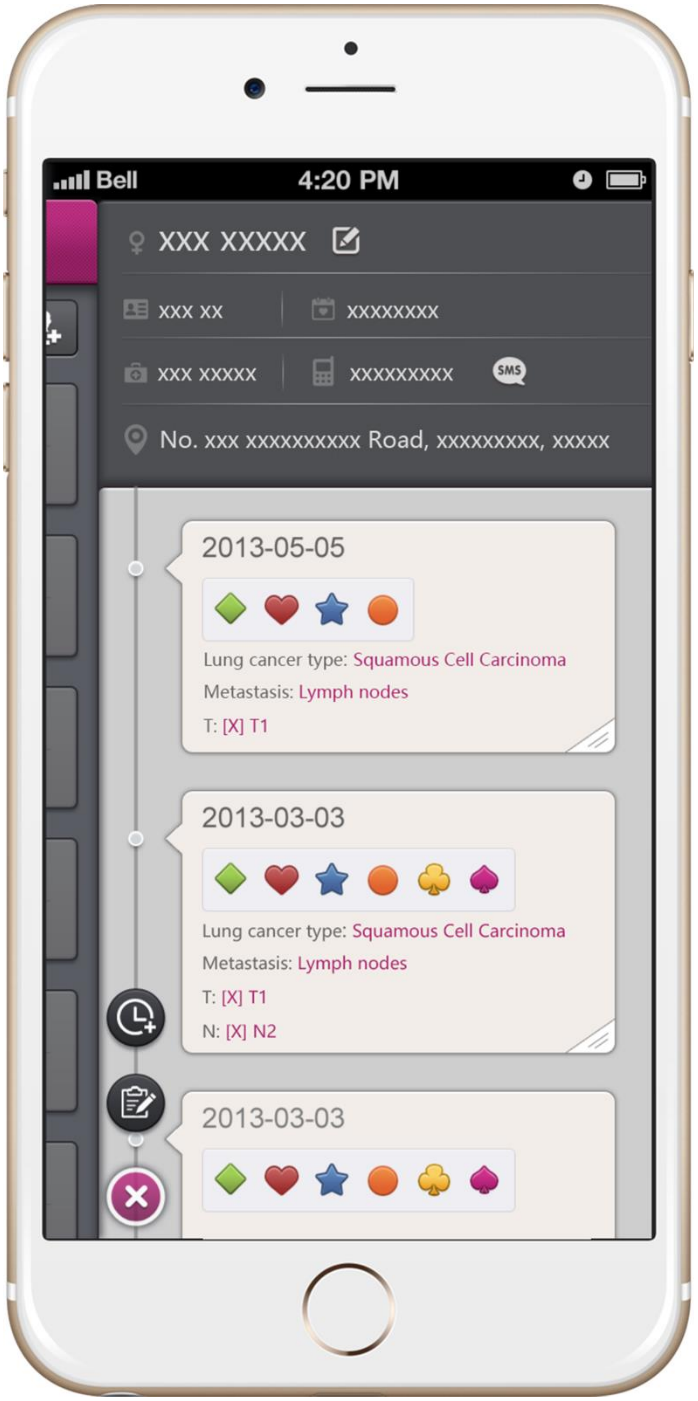

Supplementary Figure S2A: mLCCare interface on iOS

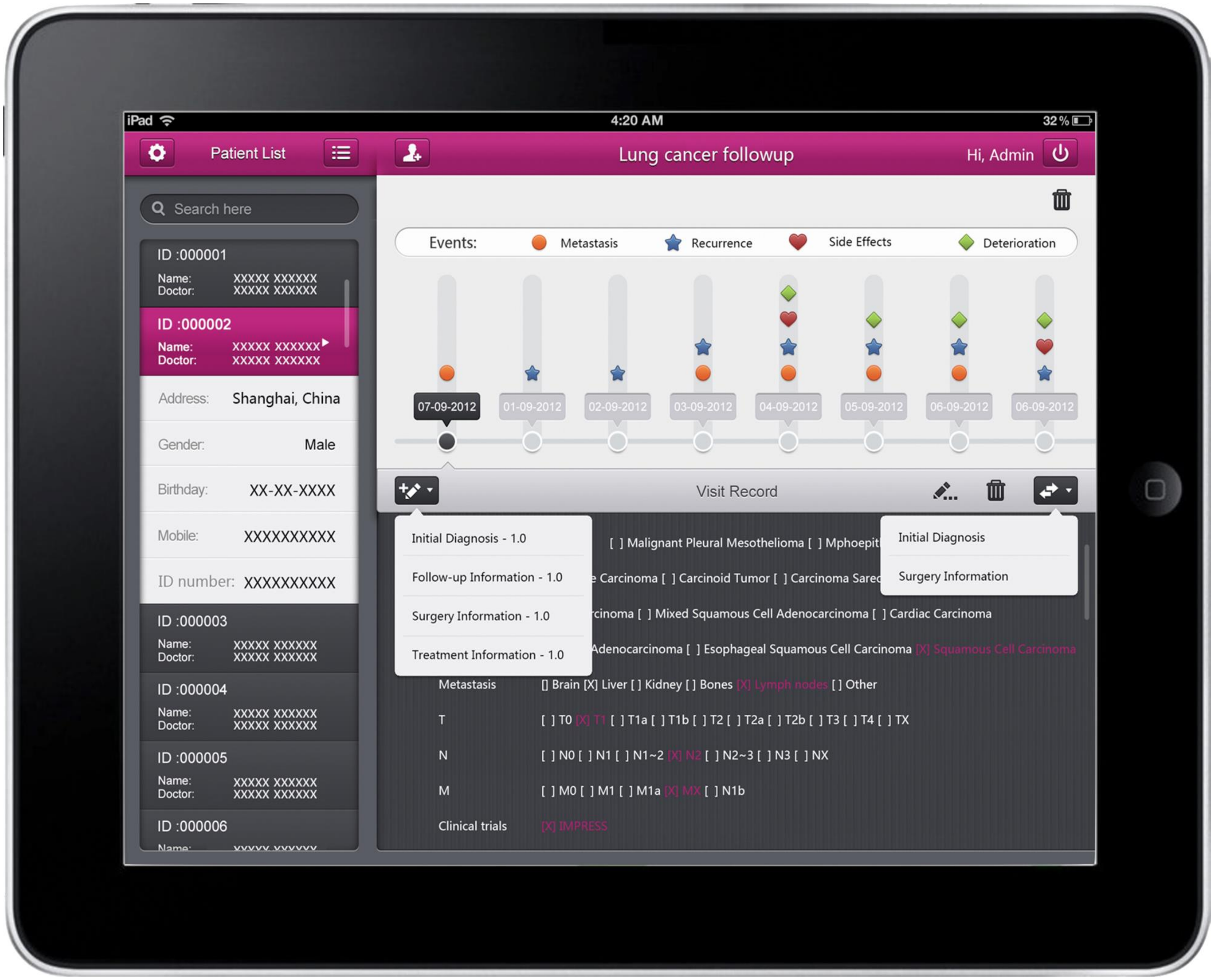

Supplementary Figure S2B: mLCCare interface on website
